# Alterations of biaxial viscoelastic properties of the right ventricle in pulmonary hypertension development in rest and acute stress conditions

**DOI:** 10.3389/fbioe.2023.1182703

**Published:** 2023-05-30

**Authors:** Wenqiang Liu, Kristen LeBar, Kellan Roth, Jassia Pang, Jessica Ayers, Adam J. Chicco, Christian M. Puttlitz, Zhijie Wang

**Affiliations:** ^1^ Stanford Cardiovascular Institute, Stanford University, Stanford, CA, United States; ^2^ School of Biomedical Engineering, Colorado State University, Fort Collins, CO, United States; ^3^ Department of Mechanical Engineering, Colorado State University, Fort Collins, CO, United States; ^4^ Laboratory Animal Resources, Colorado State University, Fort Collins, CO, United States; ^5^ Department of Biomedical Sciences, Colorado State University, Fort Collins, CO, United States

**Keywords:** pressure overload, RV failure, anisotropy, stored energy, dissipated energy, exercise capacity

## Abstract

**Introduction:** The right ventricle (RV) mechanical property is an important determinant of its function. However, compared to its elasticity, RV viscoelasticity is much less studied, and it remains unclear how pulmonary hypertension (PH) alters RV viscoelasticity. Our goal was to characterize the changes in RV free wall (RVFW) anisotropic viscoelastic properties with PH development and at varied heart rates.

**Methods:** PH was induced in rats by monocrotaline treatment, and the RV function was quantified by echocardiography. After euthanasia, equibiaxial stress relaxation tests were performed on RVFWs from healthy and PH rats at various strain-rates and strain levels, which recapitulate physiological deformations at varied heart rates (at rest and under acute stress) and diastole phases (at early and late filling), respectively.

**Results and Discussion:** We observed that PH increased RVFW viscoelasticity in both longitudinal (outflow tract) and circumferential directions. The tissue anisotropy was pronounced for the diseased RVs, not healthy RVs. We also examined the relative change of viscosity to elasticity by the damping capacity (ratio of dissipated energy to total energy), and we found that PH decreased RVFW damping capacity in both directions. The RV viscoelasticity was also differently altered from resting to acute stress conditions between the groups—the damping capacity was decreased only in the circumferential direction for healthy RVs, but it was reduced in both directions for diseased RVs. Lastly, we found some correlations between the damping capacity and RV function indices and there was no correlation between elasticity or viscosity and RV function. Thus, the RV damping capacity may be a better indicator of RV function than elasticity or viscosity alone. These novel findings on RV dynamic mechanical properties offer deeper insights into the role of RV biomechanics in the adaptation of RV to chronic pressure overload and acute stress.

## 1 Introduction

Right ventricle (RV) failure is a lethal condition that contributes significantly to the mortality and morbidity in a variety of cardiovascular diseases including pulmonary hypertension (PH), congenital heart disease, and left heart failure with preserved ejection fraction (HFpEF) (Voelkel et al., 2006; Haddad et al., 2008; Köhler et al., 2013; Konstam et al., 2018; Lahm et al., 2018). Unfortunately, there has been a lack of effective treatment for these patients, which is due in part to an inadequate understanding of the structure-function relationship of the right ventricle (RV) in physiological and pathological conditions (Konstam et al., 2018; Lahm et al., 2018). The biomechanical properties of the RV free wall (RVFW) are considered to impact its organ function (Jang et al., 2017; Liu, 2020; Liu et al., 2020), and a recent rodent study has reported a correlation between the passive elastic modulus of the RV and the end-diastolic volume (Jang et al., 2017), which is often used as an indicator of heart failure (Diebel et al., 1992; Durham et al., 1995; Cheatham et al., 1998; Zhang et al., 2014; Vonk Noordegraaf et al., 2017). Furthermore, our own ovine study showed that the RV elasticity is correlated with RV geometry and hemodynamic properties (Liu et al., 2020). These studies directly relate the RV tissue biomechanics to clinically relevant parameters, leading to a unique perspective to link the tissue biomechanics with organ function to advance the understanding of biomechanical mechanism of RV failure.

It is well known that the ventricular wall is an anisotropic and viscoelastic material (Dokos et al., 2002; Wang et al., 2016; Ahmad et al., 2018; Nguyen-Truong and Wang, 2018; Liu et al., 2021). Such behavior indicates that the tissue’s mechanical behavior is strain and strain-rate dependent, and there are energies stored and dissipated during the deformation due to the material’s elasticity and viscosity, respectively. These energy expenditure events closely affect the use of metabolic energy from cardiomyocytes during ventricular dilation and contraction in physiological conditions (e.g., at rest or under acute stress). However, most previous studies on RV biomechanics have focused on the elasticity of the tissue (Humphrey et al., 1990; Sacks and Chuong, 1993; Ghaemi et al., 2009; Holzapfel and Ogden, 2009; Valdez-Jasso et al., 2012; Hill et al., 2014; Sommer et al., 2015; Sirry et al., 2016). A few studies that reported the viscoelastic behavior of the RV have used an indentation test of the RVFW (Rubiano et al., 2016), the tensile mechanical test of neonatal RVFW (Ahmad et al., 2018) or the length-tension test of RV papillary muscles (Yamamoto et al., 1998; Stroud et al., 2002). Thus, the biaxial viscoelastic behavior of adult RVFW remains little investigated. Our group originally examined the biaxial viscoelasticity of RVFW in healthy adult sheep (Liu et al., 2021; 2022), but the changes in the viscoelastic behavior in pathological conditions are unclear. Furthermore, at the cell level or in papillary muscles, it has been shown that the myocardial viscoelasticity is increased in hypertrophy/diseased hearts and significantly weakens the muscle’s contractile function (Cooper, 2006; Caporizzo et al., 2018). Thus, the viscoelastic behavior of myocardium is critical to cardiac function. But how exactly the tissue-level viscoelasticity is altered in pathological remodeling and affects the myocardial function remains a knowledge gap.

The strain-rate dependent character of viscoelasticity determines that the ventricle wall’s mechanical behavior is different at different heart rates (HR), such as those at rest (normal HR) and under acute stress or exercise conditions (increased HR) (Schubert et al., 2009). Unlike the chronic stress that reduces the HR, acute stress caused by a psychological or physical stressor typically results in an elevated HR. The adaptation to acute stress involves changes in multiple cardiovascular parameters (e.g., cardiac output, blood pressure), which is often known as cardiovascular reactivity (Huang et al., 2013). But little is studied on the acute *mechanical* changes of the ventricle wall. The investigation of the RVFW viscoelastic changes under increased strain-rate (HR) will provide novel biomechanical insights for the RV adaptation to acute stress. Additionally, the healthy myocardium adapts to the increased workload during exercise by increasing stroke volume and heart rate, thus increasing the cardiac output—the blood supply to downstream organs. However, an impaired cardiac reserve has been noted in pulmonary arterial hypertension (PAH) and HFpEF patients, and the inability of the RV to increase cardiac output during exercise is one of key contributors (Borlaug et al., 2016; Guazzi et al., 2016; Lin et al., 2016; Jaijee et al., 2018; Malenfant et al., 2021). Whether the diseased (failing) RV has a distinct response to increased HR in its viscoelastic properties than the healthy RV is completely unknown, and the investigation of the viscoelastic changes will bring additional information to the capacity of failing RVs to adapt to the increased myocardial demand.

Therefore, the goal of this study was to investigate the alterations of RVFW biaxial viscoelastic behavior with PH development and at normal and increased heart rates simulating resting and acute stress conditions. We hypothesize that the remodeling in pulmonary hypertensive RVs leads to increased viscoelastic properties and tissue anisotropy and decreased damping capacity, and the increased HR causes a stronger reduction in damping capacity in the diseased RV than the healthy RV. This is the first study to investigate and compare the biaxial dynamic mechanical properties of the RVFW in various physiological and pathological states. The novel findings will improve our understanding of the RV tissue biomechanics in response to PH progression and increased heart rates and offer new insights into the adaptation of RV to chronic pressure overload and acute stress.

## 2 Materials and methods

### 2.1 Animal model and *in vivo* measurement

All procedures were approved by Institutional Animal Care and Use Committee (IACUC #1438) at Colorado State University. All animal experiments were performed in accordance with the guidelines and regulations of the Colorado State University Institutional Animal Care and Use Committee. All procedures were performed in accordance with the ARRIVE guidelines. Briefly, the monocrotaline (MCT) (60 mg/kg, Sigma) was injected once subcutaneously into 6-week-old male Sprague Dawley rats (Charles River) and the animals were housed in normal conditions for 3 weeks to develop PH. Similar healthy rats were used as controls (CTL). One or 2 days before euthanasia, the RV function was obtained by 2D echocardiography. Briefly, rats were anesthetized using inhaled isoflurane (0.5%–2%) and then subjected to transthoracic echocardiography using a Phillips HD11 ultrasound with a 15 mHz sector array probe. Right ventricular dimensions and contractile function including RV end-diastolic area (EDA), end-systolic area (ESA), and fractional shortening (FS) were measured in the apical four-chamber view in 2D B-mode. Pulmonary arterial (PA) outflow dynamics including peak outflow velocity (V_PA_ _peak_), outflow acceleration time (AT), ejection time (ET), and peak late (atrial) RV filling velocity (V_RV max_) were recorded using pulse wave Doppler echocardiography in the parasternal long axis (PA outflow) or apical 4-chamber view (RV filling). Prior to the mechanical tests, rats were euthanized by exsanguination after an *i.p.* injection of urethane (1.2 kg/mg) or CO_2_ inhalation. After tissue harvest, RV hypertrophy was measured by the thickness of RVFW and the Fulton index, which is the ratio of wet tissue weights calculated as RV/(LV + S), where LV is the left ventricle free wall and S is the septum (Wang and Chesler, 2013; Wang et al., 2013).

### 2.2 Specimen preparation

Fresh hearts (N = 12 for CTL and N = 10 for MCT) were obtained from CTL and MCT rats. The hearts were placed in cardioplegic solution on ice to maintain tissue viability (Witzenburg et al., 2012) and mechanically tested within 3 h after tissue harvest. The RV was dissected, and the outflow tract direction was marked as the longitudinal direction and its perpendicular direction was defined as the circumferential direction. The samples were then placed in cardioplegic solution combined with 30 mM of 2, 3-butanedione monoxime (BDM) at body temperature for at least 30 min to ensure muscle relaxation. The tissues were immersed in the same solution during the mechanical tests.

### 2.3 *Ex vivo* biaxial stress relaxation tests

After mounting, the passive equibiaxial stress relaxation tests were performed after the tissues were preloaded and preconditioned by using a newly established in-house biaxial testing system (Roth et al., 2022). Two sets of stress relaxation tests were included in this study: 1) using different ramp speeds mimicking the sub-physiological and physiological heart rates of rats (0.1, 1, 2, 5 and 8 Hz) at the fixed strain level (20%). In this study, we treated the 0.1–2 Hz testing data as sub-physiological range and the 5 and 8 Hz testing data to represent the heart rates under resting and acute stress conditions, respectively. 2) using different strain levels (6, 9, 12% and 15%) at two fixed ramp speeds (5 and 8 Hz) to reveal the type of viscoelastic behavior (linear, quasilinear or nonlinear viscoelasticity) at rest and under acute stress. Between each test, a recovery time, which was ten times that of the previous testing period, was included to ensure that the tissue was fully recovered from the previous test. Biaxial stretch forces were obtained by 5 lb load cells (Honeywell) at a sampling frequency of 200 Hz, and the engineering stress ( σ ) was calculated as the force divided by the initial cross-section area of the tissue.

**FIGURE 1:**
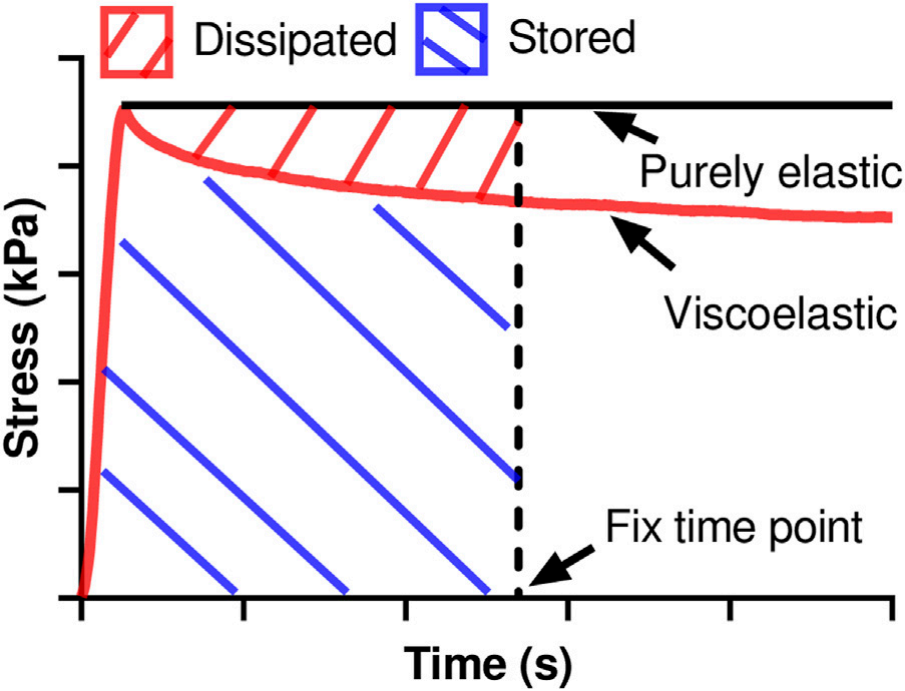
The derivations of stored energy and dissipated energy at any fixed time point use different areas calculated from the stress relaxation curve.

The relaxation modulus was derived as the stress divided by the input strain at a fixed time of the relaxation (Liu et al., 2021) and used to indicate the elastic behavior. Furthermore, from the raw data, we derived the stored energy (W_s_) as the product of the input strain and the area beneath the stress relaxation curve and dissipated energy (W_d_) as the product of the input strain and the area between the stress relaxation curve and the theoretical curve of a purely elastic material at a fixed time of the relaxation (Figure 1). We then further derived the damping capacity as W_d_/(W_d_ + W_s_) to evaluate the relative change of viscosity to elasticity during tissue deformation. The stored and dissipated energies, damping capacity, and relaxation modulus were measured at five different time points (0.01, 0.1, 1, 10, and 100 s) to indicate the viscoelastic relaxation at different stages.

Furthermore, the logarithmic scale was plotted for the stress relaxation curves obtained from the Set 2 tests. The relaxation rates and the initial stress were then derived as the slopes and the ordinate intercept of the linear fitting to the logarithmic plots at each input strain level using least square analysis. The goodness of the fit was examined by the *R* ^2^ value. The calculated relaxation rates from all strain levels were then used to determine the type of tissue viscoelastic behavior, where a fully nonlinear viscoelastic behavior is indicated if a strain-dependent behavior is observed (Troyer and Puttlitz, 2012; Liu et al., 2021; Liu et al., 2022). Lastly, the dependence of the relaxation rate or initial stress on the input strain was examined by curve fitting, and linear and nonlinear (quadratic) relations between these parameters and the input strain were determined as described previously (Shetye et al., 2014; Liu et al., 2022). The fit with a quadratic polynomial formular generated better fitting outcomes, which indicates that the RV exhibits a unique nonlinear viscoelastic behavior with a quadratic relation between relaxation rate or initial stress and the input strain.

### 2.4 Statistical and correlation analyses

Comparisons between groups (CTL and MCT), directions (longitudinal and circumferential), strain levels (6, 9, 12, and 15%), and ramp speed/frequencies (0.1, 1, 2, 5, and 8 Hz) were performed with the paired Student’s t-test or one-way ANOVA with Tukey’s *post hoc* analysis. Pearson correlation analysis was used to investigate the correlations between RV damping capacity and RV function measurement. The analyses were performed by Microsoft Excel and GraphPad Prism 9. Data are presented as mean ± SEM and *p* < 0.05 was considered statistically significant.

## 3 Results

### 3.1 RV failure establishment and structural changes in MCT RV

Transthoracic echocardiography revealed significant enlargement of RV chamber size (increased EDA and ESA) and reduced RV FS characteristic of RV dilation and systolic dysfunction, as well as marked changes in PA outflow and RV filling velocities that are characteristic of PH (lower AT/ET) and RV diastolic dysfunction (higher late V_RV max_) (Table 1). These results demonstrate the expected establishment of pathological RV remodeling in MCT rats. From the tissue measurement, we observed significant increases in the RVFW thickness and Fulton index in the MCT group compared to the CTL group, indicating RV hypertrophy in the MCT group (Table 1).

**TABLE 1:** Summary of the functional and structural measurements of the RV for both control (CTL) and monocrotaline (MCT) groups. Data are present as mean ± SEM.

Echocardiography	CTL (N = 8)	MCT (N = 8)
RV EDA (cm^2^)	0.16 ± 0.01	0.30 ± 0.03^a^
RV ESA (cm^2^)	0.09 ± 0.01	0.22 ± 0.02^a^
RV FS (%)	41.50 ± 1.66	27.09 ± 3.20^a^
AT (sec)	0.03 ± 0.002	0.02 ± 0.001^a^ (↓33%)
ET (sec)	0.07 ± 0.001	0.07 ± 0.002
AT/ET	0.40 ± 0.02	0.23 ± 0.02^a^ (↓43%)
Peak Filling Velocity (V_RV max_) (cm/s)	70.88 ± 2.79	91.00 ± 2.88^a^ (↑28%)

Structure Measurement	CTL (N = 12)	MCT (N = 10)
Wall Thickness (mm)	0.81 ± 0.05	1.19 ± 0.09^a^
Fulton Index	0.23 ± 0.01	0.56 ± 0.06^a^

^a^ < 0.01 vs. CTL.

### 3.2 Stretch-rate (frequency) dependent viscoelastic behavior of RVFW altered by PH

**FIGURE 2:**
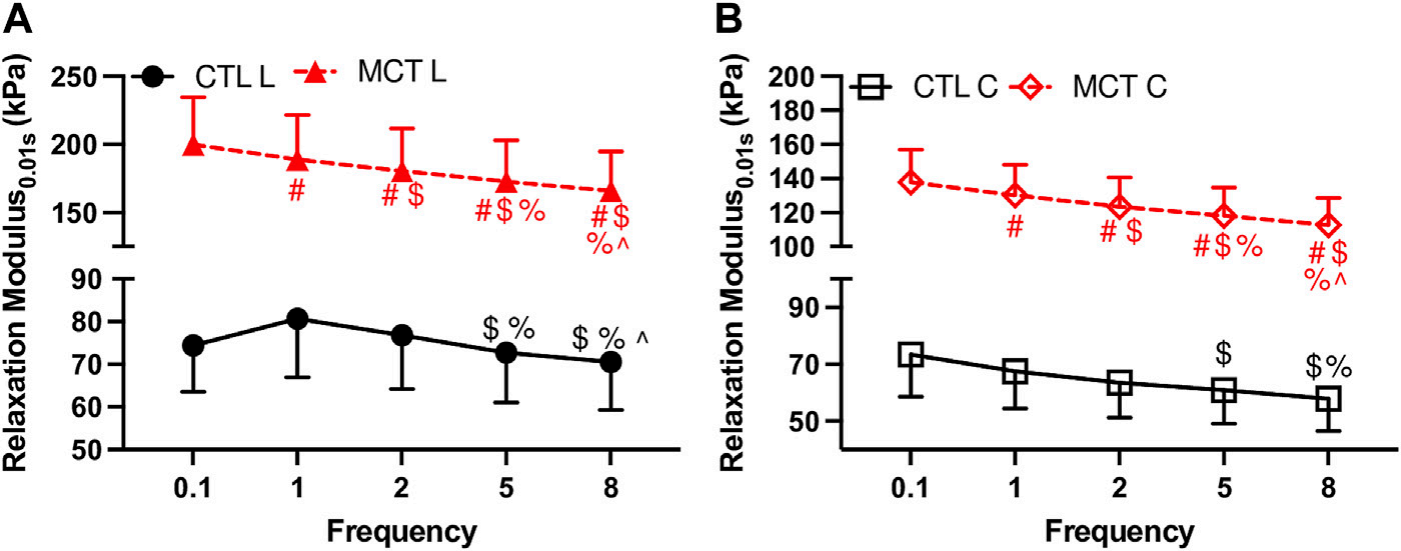
Frequency-dependent changes in the relaxation modulus at 0.01 s after peak force with an input strain of 20%. **(A)**: relaxation modulus in the longitudinal (L) direction for both groups; **(B)**: relaxation modulus in the circumferential direction for both groups. # <.05 vs. 0.1 Hz, $ <.05 vs. 1 Hz, % <.05 vs. 2 Hz, ^ <.05 vs. 5 Hz, respectively.

We firstly investigated the frequency-dependent viscoelastic behavior obtained from the Set 1 tests. Figure 2 shows significant frequency-dependent changes in the relaxation modulus measured at 0.01 s after the peak force in both groups. Firstly, the relaxation modulus curves from the MCT group were above those from the CTL group, indicating increased RV viscoelasticity in the MCT group. We further present detailed changes in the physiologically relevant conditions in the next section. Second, the MCT group presented monotonically decreased relaxation modulus with the increasing frequency in both directions, whereas the CTL group presented weaker frequency-dependent behavior than the MCT group. Therefore, PH altered the frequency-dependent viscoelastic behavior of the RV. Similar frequency-dependent behavior was observed at other time points.

### 3.3 PH increased RVFW biaxial viscoelasticity and enhanced the tissue anisotropy

**FIGURE 3:**
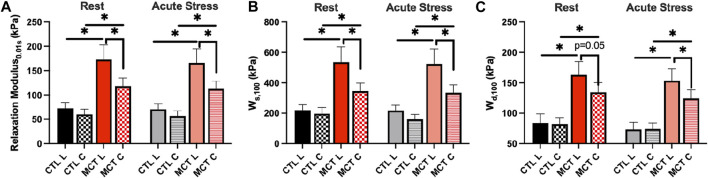
RVFW viscoelasticity before and after PH development and at resting and acute stress states. **(A)**: RV elasticity measured by relaxation modulus at 0.01 s after peak force; **(B)**: RV elasticity measured by W_S_ at 100 s after peak force; **(C)** RV viscosity measured by W_d_ at 100 s after peak force. *p <.05.

Next, we focused on the RV biaxial viscoelasticity obtained from 5 and 8 Hz with an input strain of 20% since these data are physiologically relevant. As shown in Figure 3A, we observed that the MCT RV had increased relaxation modulus than the CTL RV in both directions, and the MCT RV became anisotropic with a larger stiffness in the longitudinal than circumferential direction during PH development. Similar observation was found in the stored energy W_S_, another index of tissue elasticity (Figure 3B), and the dissipated energy W_d_, an index of tissue viscosity (Figure 3C). The same changes in these viscoelastic parameters were also observed under acute stress. Moreover, like the elastic property, the viscous property was similar between directions in the CTL RV, suggesting an isotropic type of tissue. In the MCT RV, the viscous anisotropy was pronounced in the acute stress condition (*p* < 0.05 at 0.01, 10, and 100 s, *p* = 0.058 at 1 s) and was absent in the resting condition across all relaxation stages (*p* = 0.052 at 100 s, Figure 3C). We further derived various anisotropic indices (AI) as the ratio of elastic (or viscous) parameters between longitudinal and circumferential directions (Table 2). We observed that most of the AI values were larger than 1 (except for those derived from W_d_ in CTL group), indicating a stronger elastic or viscous behavior in the longitudinal direction, and that the AI values were also larger in the elastic than viscous parameters. Moreover, the increases in AI from healthy to diseased RVs was evident only in the viscous behavior. These data indicate that PH increases RVFW elasticity and viscosity and enhances tissue anisotropy. In the diseased RV, while the elastic anisotropy was significant at both resting and acute stress conditions, the viscous anisotropy was only evident under acute stress.

**TABLE 2:** Summary of various RVFW anisotropic indices (AI) obtained from different elastic or viscous parameters in both groups and testing conditions. Data are present as mean ± SEM.

Group	AI (from relaxation modulus_0.01s_)	AI (from W_s,100_)	AI (from W_d,100_)
At Rest			
CTL	1.40 ± 0.27	1.54 ± 0.30	0.87 ± 0.11
MCT	1.45 ± 0.13	1.54 ± 0.16	1.23 ± 0.08^a^
Under Acute Stress			
CTL	1.20 ± 0.18	1.61 ± 0.30	0.88 ± 0.11
MCT	1.46 ± 0.14	1.55 ± 0.16^b^	1.25 ± 0.08^a^

^a^ < 0.05 vs. CTL in the same condition;

^b^ < 0.05 vs. resting condition of the same group.

### 3.4 PH decreased RVFW damping capacity in both directions

**FIGURE 4:**
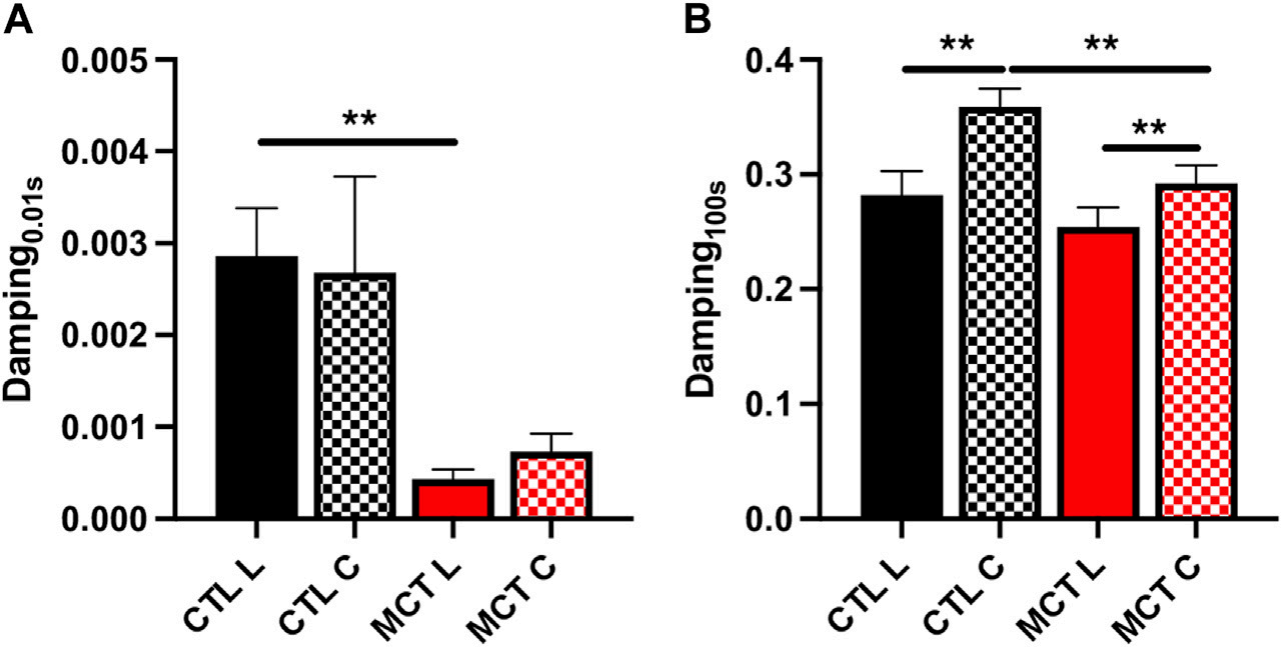
The damping capacity of the RVFW before and after PH development quantified **(A)** at 0.01 s and **(B)** 100 s after peak force. **p <.01.

We have shown previously that both elasticity and viscosity of the RV were increased with PH development, but it is unclear if these increases were equal or not. To evaluate the relative change of viscosity to elasticity, we examined the damping capacity as the percentage of dissipated (wasted) energy to the total energy during the relaxation. We found that in the longitudinal direction, the MCT RV had decreased damping capacity than the CTL RV in early relaxation (up to 1 s), and in the circumferential direction the MCT RV had reduced damping capacity in almost all stages of the relaxation (*p* <0.05 for all time points except for *p* = 0.09 for 0.01 s) (Table 3). We presented the results at 0.01 s and 100 s (equilibrium) of the relaxation obtained in normal heart rate condition in Figure 4. Similar changes were found in and the acute stress condition as well (Table 3). Therefore, this is the first report that the PH-induced RV remodeling led to reduced damping capacity of the RVFW. Lastly, we found that except for the early stage of the relaxation (at 0.01 s and 0.1 s), the RVFW damping capacity exhibited an anisotropic behavior in both rest and acute stress conditions (Figure 4B; Table 3).

**TABLE 3:** Summary of the RVFW damping capacity obtained from different groups, directions, and testing conditions. Data are present as mean ± SEM.

	Relaxation time (sec)	0.01	0.1	1	10	100
At Rest (×10^−3^)						
CTL	Longitudinal	2.86 ± 0.52	36.50 ± 5.02	118.52 ± 10.87	201.63 ± 14.64	282.01 ± 20.97
	Circumferential	2.68 ± 1.05	48.82 ± 9.51	152.18 ± 12.14^a^	258.25 ± 13.79^a^	358.81 ± 15.66^a^
MCT	Longitudinal	0.43 ± 0.10^b^	15.95 ± 2.36^b^	90.35 ± 10.15^b^	174.86 ± 13.90	254.09 ± 17.23
	Circumferential	0.73 ± 0.20	23.72 ± 3.84^a^ ^,^ ^b^	106.20 ± 8.44^a^ ^,^ ^b^	203.99 ± 12.11^a^ ^,^ ^b^	292.07 ± 15.72^a^ ^,^ ^b^
Under Acute Stress (×10^−3^)						
CTL	Longitudinal	3.54 ± 1.18	34.78 ± 7.11	118.33 ± 16.57	197.41 ± 19.13	266.88 ± 21.11
	Circumferential	3.25 ± 1.05	45.28 ± 7.33	139.27 ± 11.15	246.65 ± 13.24^a^ ^,^ ^c^	333.04 ± 14.72^a^ ^,^ ^c^
MCT	Longitudinal	0.99 ± 0.27	16.61 ± 2.76^,^ ^b^	87.69 ± 8.72	170.99 ± 12.66	247.59 ± 16.11^,^ ^c^
	Circumferential	0.85 ± 0.20^b^	24.13 ± 4.04^a^ ^,^ ^b^	103.43 ± 7.99^a^ ^,^ ^b^	198.46 ± 11.24^a^ ^,^ ^b^ ^,^ ^c^	283.99 ± 14.69^a, b, c^

^a^ < 0.05 vs. longitudinal;

^b^ <0.05 vs. CTL;

^c^ < 0.05 vs. resting condition.

### 3.5 Acute stress reduced damping capacity differently between healthy and diseased groups

**FIGURE 5:**
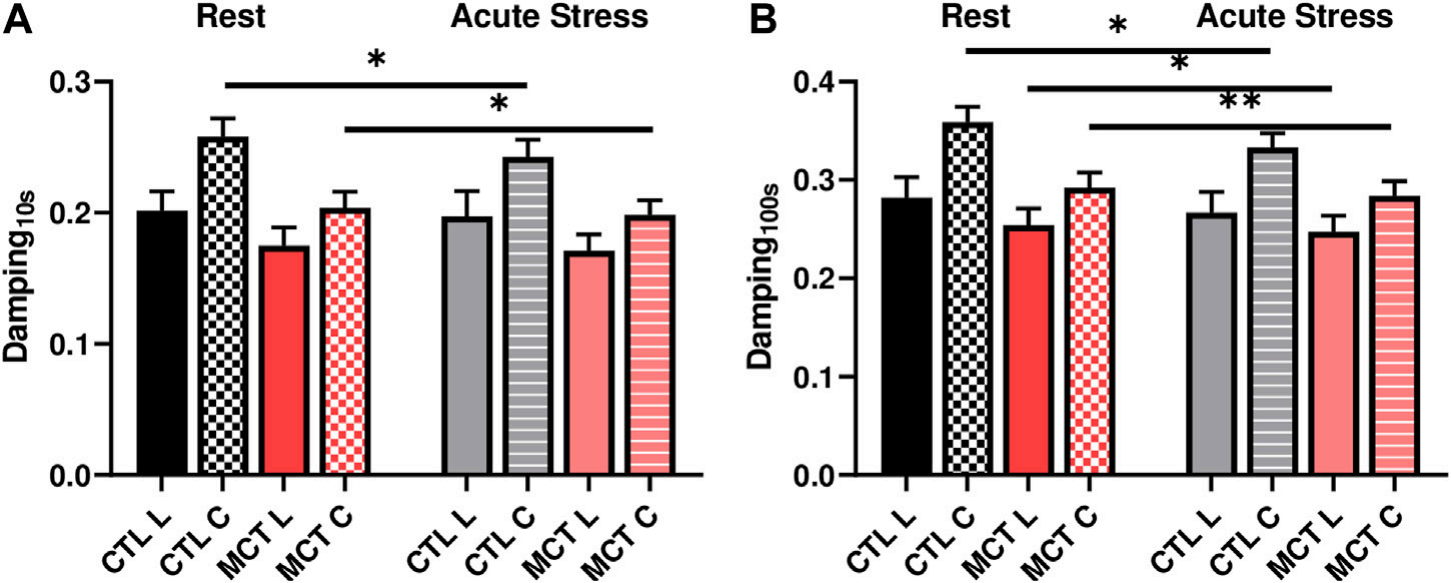
Changes in RVFW damping capacity from the resting to acute stress states at **(A)** 0.01 s after peak force and **(B)** 100 s after peak force. * <.05, ** <.01, respectively.

We further investigated the changes in the damping capacity from normal to high heart rates to reveal the viscoelastic alteration from resting to acute stress conditions. We observed that in the early stage of the relaxation (up to 1 s) there was no significant change in damping capacity in either group. At later stage of the relaxation (at 10 s or 100 s), however, the damping capacity was reduced in the tissue for both groups (Figure 5; Table 3). Moreover, the alteration was different between the CTL and MCT groups. The damping capacity was decreased only in the circumferential direction in the CTL group, whereas in the MCT group the decrease was observed in both directions and with small *p* values (Figure 5B). These data suggest that PH-induced remodeling causes a stronger reduction in damping capacity under acute stress condition than the healthy tissues.

### 3.6 Correlations between the RVFW viscoelastic properties and *in vivo* measurements

**FIGURE 6:**
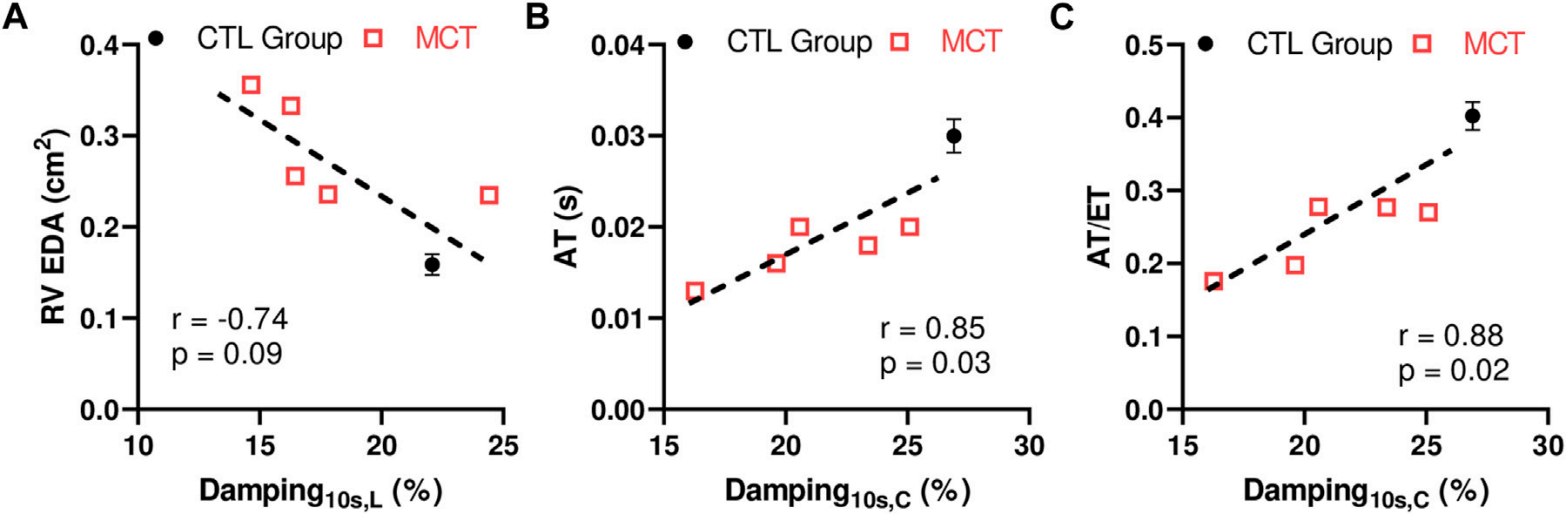
Correlations between the RVFW damping capacity at 10 s after peak force and **(A)** RV EDA, or **(B)** AT, or **(C)** ratio of AT and ET. The CTL data were included as the group average measurement (n = 8) and the error bar shows the standard error. The MCT data were included as individual data points (n = 5). L: longitudinal direction; **(C)** circumferential direction.

We further investigated the relationships between the RVFW viscoelastic parameters and the echocardiography measurements to explore potential implications of RVFW viscoelasticity. In both directions, we observed that the RVFW damping capacity tended to be strongly or was significantly correlated with the RV EDA, AT and AT/ET (Figure 6). More details of the correlations are shown in Supplementary Table S1. It is worthy of note that neither the elastic nor viscous parameter was correlated with any of these functional parameters. Our findings suggest that the damping capacity is a better indicator of RV function than the tissue elasticity or viscosity alone.

### 3.7 Altered strain-dependent viscoelastic behavior of the RVFW by PH or heart rate

**FIGURE 7:**
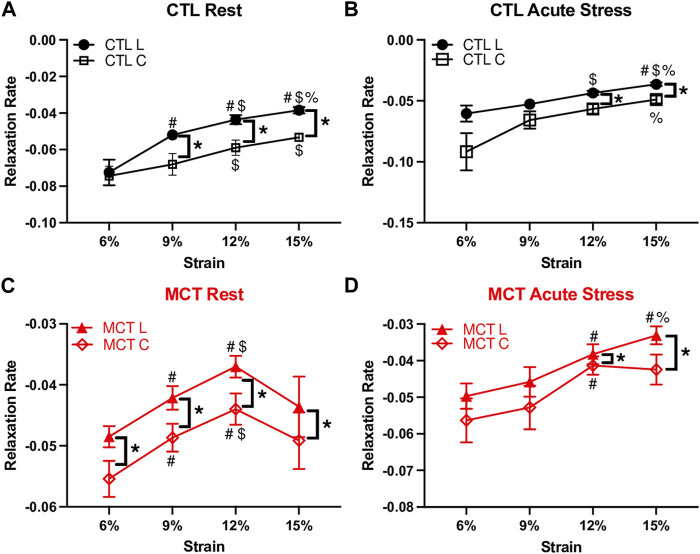
Different strain-dependent and anisotropic behaviors measured by the relaxation rate in the **(A,B)** healthy (CTL) and **(C,D)** diseased (MCT) groups in both rest and acute stress conditions. # <.05 vs. 6%, $ <.05 vs. 9%, % <.05 vs. 12%, * <.05, respectively.

From the second set of mechanical tests (i.e., stress relaxation test at varied strains), we investigated the RVFW viscoelastic relaxation altered by PH or heart rate. Specifically, we compared the rate of relaxation derived from the logarithmic plots of the stress relaxation curves. We observed significant strain-dependent behavior of the relaxation rate in both groups, and in both resting and acute stress conditions (Figure 7). This indicates that the RVFW exhibits a fully nonlinear viscoelastic (NLV) behavior in all groups and conditions. Moreover, the shape of the strain-dependent curve was different between healthy and diseased groups, between rest and acute stress conditions, as well as between directions at certain strain levels. This indicates that the RVFW has varied anisotropic NLV behaviors in different states (healthy vs. diseased, rest vs. acute stress). Therefore, it is necessary to measure the mechanical behavior of the tissue in the corresponding physiological condition.

Furthermore, we investigated the dependence of the relaxation rate or initial stress on the input strain level using either linear or quadratic curve fitting. From the relations with a good fitting (*R* ^2^ > 0.8), we found that the CTL and MCT groups had different fitting equations, indicating different NLV behaviors. Similarly, the fitting equations were different from resting to acute stress conditions, indicating that the acute stress altered the NLV behavior as well. Finally, different fitting equations were observed between directions in certain groups (e.g., the linear fit of the initial stress-strain relations at rest in the MCT group), which indicates an anisotropic NLV behavior in this group. Therefore, these data showed that the RVFW viscoelasticity exhibited different types of strain dependence in PH or acute stress conditions.

Lastly, we found that at rest, the linear relation between the relaxation rate and input strain provided good fitting to capture the NLV behavior for the CTL group (*R* ^2^ > 0.9), but it led to a poor fitting for the MCT group (*R* ^2^ = 0.29–0.42). Instead, a quadratic fitting was good to describe the relaxation rate-input strain relationship for the MCT group (*R* ^2^ > 0.9) (Table 4). These data showed that at rest, the healthy RVFW viscoelasticity had a linear dependence on the strain, whereas the diseased RVFW viscoelasticity had a nonlinear (quadratic) dependence on strain. In acute stress condition, both linear and nonlinear relations provided sufficient fitting for the CTL and MCT groups (*R* ^2^ > 0.8) (Table 4).

**TABLE 4:** The viscoelastic parameters (relaxation rate and initial stress) as a function of input strain level ( ε ). Different linear and quadratic fit equations are shown, which represent different strain-dependent viscoelastic behaviors of the RVFW in different conditions (healthy vs. diseased, rest vs. acute stress).

			Linear fit	*R* ^2^	Quadratic fit	*R* ^2^
Resting condition						
CTL	Relaxation Rate	Longitudinal	0.0037ε−0.09	0.91	$-0.0004\varepsilon^{2}+0.013\varepsilon -0.13$	0.99
		Circumferential	0.0024ε−0.09	0.99	$-0.00002\varepsilon^{2}+0.003\varepsilon -0.09$	0.99
	Initial Stress (kPa)	Longitudinal	0.54ε−2.64	0.93	$0.056\varepsilon^{2}-0.64\varepsilon +2.9$	>0.99
		Circumferential	0.50ε−2.56	0.94	$0.048\varepsilon^{2}-0.51\varepsilon +2.20$	>0.99
MCT	Relaxation Rate	Longitudinal	0.0007ε−0.05	0.29	$-0.0004\varepsilon^{2}+0.0082\varepsilon -0.085$	0.92
		Circumferential	0.0008ε−0.06	0.42	$-0.0003\varepsilon^{2}+0.0077\varepsilon -0.09$	0.95
	Initial Stress (kPa)	Longitudinal	1.03ε−3.6	0.99	$-0.0065\varepsilon^{2}+1.16\varepsilon -4.25$	0.99
		Circumferential	0.56ε−1.67	0.95	$-0.0261\varepsilon^{2}+1.11\varepsilon -4.26$	0.97
Acute stress condition						
CTL	Relaxation Rate	Longitudinal	0.0027ε−0.08	>0.99	−0.00002+0.0031ε−0.08	>0.99
		Circumferential	0.0046ε−0.11	0.91	−0.0005+0.015ε−0.16	0.99
	Initial Stress (kPa)	Longitudinal	0.50ε−2.14	0.93	$0.052\varepsilon^{2}-0.59\varepsilon +3.0$	>0.99
		Circumferential	0.49−2.42	0.93	$0.05\varepsilon^{2}-0.57\varepsilon +2.58$	>0.99
MCT	Relaxation Rate	Longitudinal	0.0019ε−0.062	0.98	$-0.0004\varepsilon^{2}+0.0011\varepsilon -0.06$	0.99
		Circumferential	0.0018ε−0.067	0.84	$-0.0001\varepsilon^{2}+0.004\varepsilon -0.08$	0.87
	Initial Stress (kPa)	Longitudinal	1.45ε−7.26	0.95	$0.125\varepsilon^{2}-1.17\varepsilon +5.11$	>0.99
		Circumferential	0.92ε−4.60	0.94	$0.08\varepsilon^{2}-0.9\varepsilon +3.9$	>0.99

## 4 Discussion

To best of our knowledge, this is the first study that investigated the changes of RVFW biaxial viscoelasticity during PH progression. The main findings are: 1) PH increased RVFW viscoelasticity in both longitudinal (outflow tract) and circumferential directions, and the tissue anisotropy was pronounced for the diseased RV, not healthy RV. 2) PH decreased RVFW damping capacity (ratio of dissipated energy to total energy) in both directions. 3) The RVFW viscoelasticity was differently altered from resting to acute stress conditions between these groups—the damping capacity was decreased only in the circumferential direction for the healthy RV, but it was reduced in both directions for the diseased RV. 4) Some correlations were observed between the damping capacity and RV function indices. 5) the RVFW exhibited a fully NLV behavior, and this behavior was altered by disease progression (PH) or heart rate. These findings will improve the understanding of RV tissue dynamic mechanical behavior with PH development and under acute stress and shed light onto the biomechanical mechanism of RV failure and the reduced exercise capacity of failing RVs.

### 4.1 PH increased RVFW viscoelasticity and enhanced RVFW anisotropy

The hemodynamic measurement clearly demonstrated that RV failure was established in the MCT rats (Table 1). Signs of RV failure was further evidenced by the dilation and hypertrophy in the RVs of the MCT group (Table 1). With PH development, we found significant changes in the biaxial viscoelasticity of RVFW. In terms of the elasticity, we observed increases in elasticity in both directions, with more increase in the longitudinal than circumferential direction (Figures 3A, B). Thus, it led to an enhancement in the anisotropic, elastic behavior of the RV (*p* <0.05). In terms of the viscosity, we observed similar increases (Figure 3C) and an altered anisotropy (from more viscosity in circumferential direction to more viscosity in longitudinal direction, Table 2) during PH development. PH also led to a stronger anisotropy in elasticity from resting to acute stress condition (Figure 3; Table 2).

The increase in myocardium viscoelasticity during heart failure progression (including the RV failure) has been reported previously. Typically, 1D force/tension-length measurements have been performed in isolated cardiomyocytes or papillary muscles, and the viscoelastic property has been quantified from stress relaxation, cyclic stress-strain hysteresis, or the loading stress-strain curve (Dokos et al., 2002; Linke and Fernandez, 2002; Stroud et al., 2002; Cooper, 2006; Caporizzo et al., 2018; Caporizzo et al., 2020). Increases in elasticity and viscosity of the diseased myocardium were noted in these studies. But these methodologies assume an isotropic material of the myocardium and are mainly restrained at the cell level. Rubiano et al. (2016) induced systemic hypertension (not pulmonary hypertension) in adult rats and both left and right ventricles (LV and RV) were hypertrophied. They measured the ventricular tissue viscoelasticity by indentation tests (assuming isotropic material) and a standard linear viscoelasticity model. Interestingly, the RVFW showed increased elasticity and viscosity in the hypertensive rats, whereas the LVFW had only increased viscosity. Thus, our observation of increased elasticity and viscosity in the RVFW is consistent with prior findings in failing hearts. More importantly, the present study provides for the first time the tissue level, anisotropic viscoelastic findings of the RVFW and fills a critical gap of knowledge.

Not surprisingly, we observed increases in stiffness in both longitudinal and circumferential directions, and the increase was more significant in the longitudinal direction. This led to a change of tissue anisotropy and the RVFW became anisotropic (Figure 3). Similar observations have been reported previously when hyperelastic properties of the RVFW is examined by other groups. The enhanced stiffening in the longitudinal direction has been attributed to the re-orientation of the myofibers toward the long axis during PH progression, and the fiber re-orientation contributes to the changes in both RV diastolic and contractile function (Hill et al., 2014; Avazmohammadi et al., 2017a; Avazmohammadi et al., 2017b; Avazmohammadi et al., 2019). Therefore, our observation in RVFW elasticity agrees with prior findings. Moreover, we observed similar increases in viscosity (W_d_) in the diseased RVFWs, and the viscous anisotropy was stronger in the acute stress than resting condition. These elastic and viscous changes in the diseased RVFW may be related to several different molecular mechanisms contributing to these mechanical properties. In the PH-induced remodeling, the RV hypertrophies (Kuroha et al., 1991; Heitmeier et al., 2020; Nguyen-Truong et al., 2020) and collagen accumulation occurs (Lahm et al., 2018; Bogaard and Voelkel, 2019; Poble et al., 2019; Nguyen-Truong et al., 2020). Both increases in intracellular microtubules (a cytoskeleton that confers myocyte mechanical strength) and extracellular collagen fibers are expected to stiffen the material property of the tissue in all directions. In addition, it is well accepted that the tissue is the stiffest in the main fiber direction. When the myofibers and collagen fibers get re-aligned and closer to the longitudinal (outflow tract) direction, the RVFW becomes more anisotropic and stiffer in this direction as well. But the molecular mechanism of viscosity is much less known. The myocardial tissue viscosity depends on the sources of frictional forces, which include diverse intra-and inter-molecular frictions—from cytoskeletons (e.g., microtubules) of cardiomyocytes to extracellular matrix proteins (e.g., collagen, proteoglycans) and even interstitial fluids. To the best of our knowledge, there is no known dominant “direction” of frictional sliding in aligned fibrous materials. Indeed, compared to the elastic anisotropy, viscous anisotropy is rarely reported in aligned biomaterials. Our tissue data here seem to suggest that there was weaker anisotropy in viscosity in the remodeled RV in the resting condition, and the increased heart rate (acute stress condition) enhanced the viscous anisotropy (Figure 3B). Since viscosity is related to the energy dissipation due to weak molecular bonds, we speculate that the dynamic distribution of external bonds between collagen fibers (e.g., inter-fiber crosslinking) and/or myofibers is responsible for the observations in the diseased RV. Future work should elaborate the exact molecular mechanisms of the viscosity from various sources such as fiber and non-fiber components.

Lastly, since both elasticity and viscosity were increased in diseased RV, we examined the relative change of viscosity to elasticity by quantifying the damping capacity (W_d_/(W_d_ + W_S_)). We observed the reduction of damping capacity in both directions (Figure 4). But the reduction was significant only in the early relaxation (up to 0.1 s) in the longitudinal direction and was significant in the most stages of relaxation in the circumferential direction (Table 3). This is observed both in the resting and acute stress conditions. These data suggest that the changes of viscosity and elasticity are not “matched” and the increase in elasticity is more pronounced than that in viscosity in PH development. It is accepted that the stiffer myocardium results in elevated wall stress and it is detrimental to the cardiomyocytes. But the role of viscosity in myocardium physiology is less clear. On one hand, the higher viscosity of the diseased tissue could result in more energy waste; but on the other hand, it may protect the cells by damping the stress. Moreover, it has been shown from cell mechanotransduction studies that the viscous substrate reduces the cell’s focal adhesion area due to a dissipation (loss) of tractional force and cytoskeletal tension, thus producing enhanced cell spreading (morphology) and the associated mechanosensing pathway (Curtis and Russell, 2009; Ryan and O’Brien, 2015). Therefore, the concomitant changes in myocardial elasticity and viscosity may be driven to maintain a mechanical homeostasis for the cardiomyocytes. However, in the failing RVs, the homeostasis (unmatched elasticity and viscosity) cannot be preserved and such disturbance may be responsible for the cellular dysfunction and organ failure.

### 4.2 Altered response of the diseased RV to acute stress

In the present study, we exposed the RVFW to acute stress condition by increasing the stretch rate and examined the viscoelastic response of the tissue. Our results showed that when changed from the resting to acute stress conditions, the healthy RVFW had decreased damping capacity only in the circumferential direction, whereas the failing RVFW had decreased damping capacity in both directions (Figure 5). Therefore, the healthy RV may be better in handling acute stress in terms of maintaining the mechanical homeostasis. Moreover, the unchanged damping capacity in the longitudinal direction in the healthy RV suggests that the mechanical energy waste in the main blood flow direction is preserved. In contrast, the diseased RV had more significant reduction in damping capacity in both directions, suggesting the tissue has poor response to manage the acute stress. It has been noted recently that the heart failure patients, including the RV failure patients, have reduced exercise tolerance (Borlaug et al., 2016). That is, the exercise challenge imposes a negative impact on the RV function and the dysfunctional RV has poor cardiac reserve for exercise (Borlaug et al., 2016). In response to the acute stress such as exercise, the ventricle typically contracts faster and stronger, thus leading to higher heart rate and stroke volume to increase the blood supply and meet the elevated metabolic demand. However, a clinical study showed that in the RV dysfunction patients with reduced exercise capacity, despite an increased heart rate, the increase in cardiac output was limited due to a weaker contractility (Guazzi et al., 2016). Therefore, our finding offers a mechanical explanation of the impaired exercise capacity in failing RVs: the healthy RVs adapt to the acutely increased heart rate by preserving the mechanical homeostasis along the main blood flow direction, whereas the diseased RVs failed to do so. Our data suggest that the damping capacity may be a critical viscoelastic parameter of the RV biomechanics, and we should include this viscoelastic property of the myocardium into consideration to fully understand the role of RV biomechanics in maladaptive remodeling.

### 4.3 Altered strain-dependent viscoelastic behavior of RV by PH or acute stress

Linear, quasi-linear and fully nonlinear are the three main types of viscoelastic behavior. While all these three behaviors are time-dependent, the linear viscoelastic material exhibits linear elastic and linear viscous behavior, whereas the quasi-linear viscoelastic material exhibits nonlinear elastic and linear viscous behavior. The “linear viscous behavior” can be revealed by showing the same shape of relaxation or creep curves at different input strains. That is, the time-dependent behavior is *strain-independent* or the viscosity and elasticity are separable (Lakes, 1998; Troyer and Puttlitz, 2011). In contrast, in a fully nonlinear viscoelastic (NLV) material, both elasticity and viscosity are *strain-dependent* and they are not separable. Our results showed that the RV viscoelastic behavior was nonlinear viscoelastic (NLV) in different states (physiological or pathological) and at different heart rates (at rest and acute stress). This means that for both healthy and diseased RVs, the tissue’s elasticity and viscosity are not separable. Moreover, we observed different shapes of the strain-dependent relaxation rate curves between healthy and diseased groups, between the two axes and between resting and acute stress conditions (Figure 7). These differences indicate that PH or acute stress (increased heart rate) altered the RV anisotropic NLV behavior, and it is important to obtain the tissue’s dynamic mechanical behavior in the corresponding physiological condition.

### 4.4 Implications of RV viscoelasticity in RV function

In the present work, we performed some preliminary work to unravel the implications of the RV viscoelasticity in its physiological performance. The correlation between the passive elastic modulus of the RV in the longitudinal direction and the end-diastolic volume has been reported in a rodent study (Jang et al., 2017). Similarly, correlations between the RV longitudinal elasticity and RV end-diastolic diameter/area were found by our group in a recent large animal study (Liu et al., 2020). Compared to the RV elasticity, the RV viscoelasticity is much less studied. As a result, whether and how the RV viscoelasticity is associated with the RV function is still unknown. Our results showed some preliminary correlations between the RV damping capacity and the *in vivo* parameters obtained from echocardiography (Figure 6); however, these correlations were absent for the RV elasticity indices (relaxation modulus and stored energy) and nearly absent for the RV viscosity indices (dissipated energy and relaxation rate). The discrepant results may be because of the different types of mechanical tests and/or testing conditions. The previous studies derived the tissue elasticity from the loading curve of cyclic tensile mechanical tests and using a sub-physiological stretch rate (quasi-static) (Jang et al., 2017; Liu et al., 2020), whereas the RV elasticity within this study was derived from the stress relaxation with a ramp speed similar to the diastolic stretch rate in rat hearts. Nevertheless, our data highlight the necessity to obtain the dynamic mechanical behavior under physiological conditions when assessing RV performance.

Furthermore, we reported a strong trend of correlation between RV damping capacity and RV EDA. Increase in RV end-diastolic size is closely associated with disease severity and mortality of the RV failure patients (Prihadi et al., 2019; Lejeune et al., 2020; Ji et al., 2022). Thus, our result indicates that the RV damping capacity may potentially indicate the severity of the RV dysfunction. Our results also showed that the damping capacity was strongly correlated with the AT of pulmonary outflow. Preclinical studies of PH subjects has reported a correlation between the ratio of systolic pulmonary artery pressure to AT and pulmonary vascular resistance (afterload) (Thibault et al., 2010). Thus, AT and AT/ET are used to non-invasively estimate RV systolic pressure to indicate the severity of RV dysfunction. The additional correlations between AT or AT/ET and damping capacity found in this study suggest again the role of damping capacity in predicting the severity of RV dysfunction.

### 4.5 Limitations

All the data were obtained from male rat RVs, and there might be a sex difference in the RV viscoelastic behavior that awaits future research. In this study, we mainly used increased heart rate (thus higher stretch speed at diastole) to mimic the blood filling in the acute stress condition and characterize the passive mechanical properties. However, it is possible that the maximal strain is also increased during acute stress as a result of larger venous blood return. We assume the change in the maximal strain is not significantly as the prior study showed an increase of heart rate from resting to acute stress conditions but the same stroke volume in pulmonary arterial hypertension patients (Groepenhoff et al., 2010). Furthermore, the sample size for our correlation analysis is small, and we used the group average value for the CTL group to estimate the correlations. Future work should confirm the correlations with more samples and using individual values.

### 4.6 Conclusions

To the best of our knowledge, this is the first study on the changes of RV biaxial viscoelastic behavior during PH development. Our results from the *ex vivo* stress relaxation tests showed that PH increased RV viscoelasticity in both directions. These changes resulted in an anisotropic viscoelastic behavior of the diseased RV with marked elastic anisotropy in both resting and acute stress conditions and the viscous anisotropy only in acute stress condition. PH development also reduced the damping capacity (ratio of dissipated energy to total energy) of the tissue in both directions. In addition, the RV viscoelasticity was acutely altered from resting to acute stress conditions—the damping capacity was decreased only in the circumferential direction for healthy RVs, but it was decreased in both directions for diseased RVs, indicating an impaired response to acute stress. Lastly, the RVFW exhibited a fully NLV behavior and this behavior was altered by disease progression (PH) or heart rate. These novel findings improve our understanding of RV biomechanics in response to chronically elevated pulsatile mechanical loadings or acutely increased heart rates, and they will shed light on the mechanical mechanism of RV failure and the reduced exercise capacity.

## Data Availability

The original contributions presented in the study are included in the article/Supplementary Material, further inquiries can be directed to the corresponding author.
