# Supplementary material for: Alterations of biaxial viscoelastic properties of the right ventricle in pulmonary hypertension development in rest and acute stress conditions
Source: Front Bioeng Biotechnol. 2023 May 30;11:1182703. doi: 10.3389/fbioe.2023.1182703 (PMC10266205; doi:10.3389/fbioe.2023.1182703)
Supplement: Supplementary file 1 [file Table1.docx]

**Supplemental Material**

**Table 1.** The p values of correlations between the RVFW damping capacity and echocardiography measurements. The damping capacity was obtained at various time points of the relaxation after the peak stress in the longitudinal (L) and circumferential (C) directions.

| **Damping Capacity** |  | **RV EDA (cm^2^)** | **RV ESA (cm^2^)** | **RV FS (%)** | **V_PA peak_ (cm/s)** | **AT**  **(sec)** | **ET**  **(sec)** | **AT/ET (%)** | **V_RV max_ (cm/s)** |
| --- | --- | --- | --- | --- | --- | --- | --- | --- | --- |
| At 0.01s | L | NS | NS | NS | NS | * | NS | * | * |
|  | C | NS | NS | NS | NS | ** | NS | * | p = 0.05 |
| At 0.1s | L | * | NS | NS | NS | * | NS | * | NS |
|  | C | NS | NS | NS | NS | ** | NS | * | * |
| At 1s | L | * | NS | NS | NS | NS | NS | NS | NS |
|  | C | NS | NS | NS | NS | * | NS | * | NS |
| At 10s | L | NS | NS | NS | NS | NS | NS | NS | NS |
|  | C | NS | NS | NS | NS | * | NS | * | NS |
| At 100s | L | NS | NS | NS | NS | NS | NS | NS | NS |
|  | C | NS | NS | NS | NS | * | NS | * | NS |

**^*^** < .05, **^**^** < .01, respectively. NS: no significant correlation.
